# Rethinking Growth Monitoring and Promotion: Is It Time for A New Approach?

**DOI:** 10.1016/j.advnut.2024.100363

**Published:** 2024-12-30

**Authors:** Jo-Anna B Baxter

**Affiliations:** 1 Department of Nutritional Sciences, University of Toronto, Ontario, Canada; 2 Centre for Global Child Health, Hospital for Sick Children, Ontario, Canada

Although child survival has increased, child undernutrition remains a prevalent global health issue. Young children in low- and middle-income countries (LMICs) are at increased risk of growing slower than expected rates (i.e. growth faltering), including underweight, stunting (linear growth restriction), and wasting [1]. Worldwide, an estimated 148 million children under 5 y of age (22.3%) are affected by stunting and 45 million (6.8%) by wasting [2]. The consequences of growth faltering can range from increased infection susceptibility and compromised development to, in the severest cases, mortality [3]. Growth faltering presents a persistent and unresolved global challenge, and comprehensive solutions are needed. Growth monitoring and promotion (GMP) serves as one component within the larger picture of support for child health and nutrition.

Monitoring child growth has long been used as a diagnostic tool for overall health and well-being. Ashworth et al. [4] helpfully detail the history and development of growth monitoring programs. Briefly, there is documentation of the use of sequential measurement of growth in infants since the 1800s, with the first known growth references in the early 1900s [5]. The use of growth charts has been recommended by the WHO and the Food and Agricultural Organization since the 1960s [6], and international, multisite growth standards and charts are used around the world today [7]. However, the effectiveness of growth monitoring in addressing child undernutrition in LMICs has been a matter of debate for over 40 y [4]. This has largely related to concerns about coverage and measurement quality, and limitations within already constrained health systems to adequately promote growth. UNICEF came to champion GMP in the 1980s as a key component of their primary health care strategy, which also included oral rehydration, breastfeeding, and immunization. GMP includes taking routine anthropometric measurements (e.g. weight, length/height monthly from 0 to 24 mo of age); assessing the adequacy of growth relative to an appropriate standard; and offering nutrition promotion (i.e. counseling and other services to improve nutrition and growth). The term GMP was purposefully chosen to emphasize the importance of growth-promoting activities, because they could be overshadowed by anthropometric measurements and charting [8]. The discussion of GMP continues today, with a recent systematic review (6 studies) concluding that there is limited uncertain evidence on the effectiveness of GMP programing in LMICs on key health indicators (i.e. child growth faltering, feeding practices, health service utilization) [9].

In this issue of *Advances in Nutrition*, Leroy et al. [10] critically examine the epidemiological basis of GMP—whether GMP can accurately diagnose or identify children with healthy compared with inadequate growth. They argue that different GMP criteria (e.g. weight-for-age *z*-score below −2) do not accurately predict growth later in childhood, and that collecting weight and length/height measures does not adequately identify which children need or will benefit from interventional support. To illustrate their point, they comprehensively reviewed GMP guidance documents, including documents from UNICEF, the WHO, and 7 countries with strong programs; assessed growth trajectories among a cohort of healthy children; and applied commonly used GMP criteria to see if inadequate growth could be predicted in individual children from rigorous studies conducted in LMICs. Across the board, the authors noted fluctuations in growth and that GMP criteria did not determine future childhood growth outcomes well.

As acknowledged by Leroy et al. [10], measuring child growth is challenging, and a strength of their analysis is the use of data from multiple large-scale research studies. This offers higher quality data than routine GMP given the standardization of measurement collection. Nonetheless, collecting child anthropometric data are important to monitoring national and regional trends and disparities in malnutrition. There continues to be a need to measure child growth at a population level in LMICs (e.g. Demographic Health Surveys) to inform advocacy and facilitate policy change, as well as guide public health interventions. Among nongovernmental agencies, there is also a need demonstrate the impact of implemented programming, with growth measures often used to assess the effectiveness of health and nutrition programs. Leroy et al. [10] also acknowledge that monitoring children’s nutritional status is important to the justification of programming and intervention targeting, and suggest surveying a sample of children.

Two complexities continue to affect GMP. The first is that the frequent anthropometric measurement component of GMP continues to overshadow growth promotion efforts in the first year of life—although growth monitoring is acknowledged as an entry point for nutrition counseling and age-appropriate guidance related to breastfeeding and complimentary feeding [4]. Capacity and guidelines for implementing effective growth promotion remain a challenge in LMICs. The second complexity is the tension between considering a child as an individual (i.e. personalized nutrition interventions) compared with a member of a population (i.e. community-wide interventions). Besides wasting, which necessitates individual-based treatment and is affected by proximal factors (e.g. illness, lack of food), many interventions in LMICs cannot be delivered practically on an individual level (e.g. constrained health system, resource limitations). Moreover, linear growth faltering has been characterized as a condition that affects populations [11], necessitating multisectorial, population-level interventions to address underlying root causes (e.g. poverty, community-level food insecurity).

Thinking more broadly, growth starts from conception. Improving a child’s growth does not simply start with the child. Growth starts from conception and requires a life course approach, with geographical tailoring [3]. Addressing stunting and wasting should include improving existing interventions for women of childbearing age, as well as greater emphasis on interventions for children under 6 mo of age, as this period is currently underexplored and could be a window of opportunity [12,13]. An important knowledge gap identified by Leroy et al. [10] is our understanding of the environment in which children grow and develop (e.g. available and accessible nutrient-rich foods, sanitation, access to high-quality health care). Although there is a tendency to focus on child growth as an exposure for later complications, rethinking children’s environment could be helpful and better address the underlying causes of malnutrition.

We currently find ourselves over halfway through the Sustainable Development Goal era, which includes the ambitious goal of ending malnutrition by 2030 [14]. The tools available to those in the global nutrition field have long been critiqued, yet a lack of consensus as to the way forward persists. What has not changed is the need to invest in the health and well-being of children in LMICs. Making meaningful progress will require engaging diverse stakeholders (e.g. governments, organizations, researchers, practitioners) to refine growth monitoring practices, and ensuring corresponding evidence-based growth promotion strategies—including addressing underlying causes of malnutrition. A unified effort is required to ensure a healthier future for all children.

## Author contributions

The sole author was responsible for all aspects of this manuscript.

## Funding

The authors reported no funding received for this study.

## Conflicts of interest

The author reports no conflicts of interest.
